# The training of otorhinolaryngologists in Brazil

**DOI:** 10.1016/S1808-8694(15)30139-7

**Published:** 2015-10-18

**Authors:** Agricio Crespo

**Affiliations:** Head of the Otorhinolaryngology Department of the School of Medical Sciences of the University of Campinas-UNICAMP; Coordinator of the Teaching, Training and Residency Committee of the ABORL-CCF

The training of otorhinolaryngologists can happen in medical residency programs certified by the Ministry of Education and Culture (MEC), with a right to the title of specialist immediately after completing the program, or in specialization programs certified by the Brazilian Association of Otorhinolaryngology-Neck and Facial Surgery (ABORL-CCF), which give the title of specialist to those who pass a test from the latter institution afterwards.

Specialization and medical residency programs are defined as graduate studies for physicians and are characterized as “on the job training”, with theory and practice, at a full time basis, carried out in public or private institutions, universities or not, under the supervision of highly qualified and ethical professionals.

The program certification is based on fulfilling the minimum requirements established by MEC or those from the ABORL-CCF, and the latter is more stringent in terms of the program”s content. The criteria established by MEC are more stringent in terms of the legal aspects of teaching, with the mandatory granting of a scholarship, a certain number of hours the resident has to attend the service per week, vacations and others.

These requirements aim at guaranteeing the desirable profile of the ENT specialist, which is the goal to be reached at the end of training, based on a set of skills and knowledge that student has to acquire during training, providing them the expertise to properly perform as specialists after they are finished with the program.

The Committee of Higher Level Professional Improvement - Comissão de Aperfeiçoamento Profissional de Nível Superior (CAPES) assesses and classifies the sensu strictu graduate programs through a stringent and acknowledged process which has continuously improved the quality of research in Brazil.

The National Committee of Medical Residency Programs- Comissão Nacional de Residência Médica (CNRM) is yet to develop such a comprehensive evaluation system, requiring medical residency programs to fulfill the “minimum set of criteria”. CNRM criteria level off the teaching programs through minimum parameters which; however, do not stress quality nor foster their improvement.

The spots available for medical residency in otorhinolaryngology offered by medical schools in universities do not match the demand medical students have for this specialty. Approximately 50% of the candidates enrolled to take the test for the title of specialist offered by the Brazilian Association of Otorhinolaryngology – Neck and Face Surgery are candidates coming from training programs developed in general hospitals or private clinics. These training programs are numerically significant to the training of Brazilian otorhinolaryngologists, considering the profile of this specialty in our country. Of these, some are certified by MEC and others, by ABORL-CCF. Since they are not being held in an academic environment and, eventually, are led by program coordinators without formal academic training, often times their attendees may be exposed to insufficient practices in the without the proper content supervision.

It is very likely that there are residency programs which are not fully integrated in the training activity, prioritizing medical care and service providing. In extreme conditions, maybe even not so rare, the residents could act as mere service providers, low cost workers, in exchange for some badly-qualified learning opportunity.

The institution”s reputation, nor even that of its members, represents full guarantees of the teaching quality it provides.

How do medical residency programs in Brazil compare to those from developed countries? The lack of accuracy in current information does not allow us to make such comparison, contrarily to what happens with sensu strictu programs.

In 2004, the ABORL-CCF developed and implemented a robust, original and pioneer model to assess medical residency programs in Otorhinolaryngology in Brazil. The programs were then classified as A, B+, B, C+, C, D and E.

The existence of an assessment process, based on specific and well defined criteria, considering comprehensive quantitative and qualitative aspects of the technical, scientific and ethical training, created conditions for the continuous self-assessment of the programs by their supervisors and by the residents themselves. Programs which were knowingly deficient in specific areas could establish cooperation agreements with others as a means of educational complementation for the deficient program. The identification of these drawbacks provided subsidies for the improvement of deficient programs, fostered the development of quality goals and disqualification of those programs that would not be able to reach them.

The Assessment and Classification Process of Otorhinolaryngology Medical Residency Programs - Programa de Avaliação e Classificação dos Programas de Residência Médica e Especialização em Otorrinolaringologia (PACRE) has celebrated its fourth year of existence. It has influenced the new MEC assessment criteria and is being adopted by other medical specialties in Brazil and abroad.

It is time to assess its results, limitations and impact on the improvement of Otorhinolaryngology training in Brazil.

